# Comparative analysis of miRNA-mRNA interaction prediction tools based on experimental head and neck cancer data

**DOI:** 10.31744/einstein_journal/2025ao1372

**Published:** 2025-04-07

**Authors:** Bárbara dos Santos Dias, Larissa Figueiredo Alves Diniz, Lucca D’Arco Corrêa, Rafael Pereira de Souza, Leticia Torres Ferreira, Denise da Cunha Pasqualin, Rafael de Cicco, Eloiza Helena Tajara da Silva, Patricia Severino

**Affiliations:** 1 Hospital Israelita Albert Einstein São Paulo SP Brazil Hospital Israelita Albert Einstein, São Paulo, SP, Brazil.; 2 Universidade de São Paulo Programa de Pós-Graduação Interunidades em Biotecnologia São Paulo SP Brazil Programa de Pós-Graduação Interunidades em Biotecnologia, Universidade de São Paulo, São Paulo, SP, Brazil.; 3 Universidade Estadual Paulista "Júlio de Mesquita Filho" São Paulo SP Brazil Universidade Estadual Paulista "Júlio de Mesquita Filho", São Paulo, SP, Brazil.; 4 Instituto do Câncer Dr. Arnaldo Vieira de Carvalho São Paulo SP Brazil Instituto do Câncer Dr. Arnaldo Vieira de Carvalho, São Paulo, SP, Brazil.; 5 Faculdade de Medicina de São José do Rio Preto São Jose do Rio Preto SP Brazil Faculdade de Medicina de São José do Rio Preto, São Jose do Rio Preto, SP, Brazil.

**Keywords:** Squamous cell carcinoma of head and neck, MicroRNAs, Biomarkers, Gene expression, Target prediction tool, Computational biology, Algorithms

## Abstract

**Objective:**

Head and neck squamous cell carcinoma (HNSCC) has a poor prognosis largely due to late diagnosis and a lack of reliable biomarkers. MicroRNAs (miRNAs), small non-coding RNAs that regulate gene expression, are promising biomarkers for HNSCC. This study evaluated miRNA-mRNA interactions in HNSCC using conventional computational tools and validated the results using molecular data.

**Methods:**

We compared three miRNA-mRNA interaction prediction tools, TargetScan, miRDB, and miRWalk, using differentially expressed miRNAs and mRNAs from HNSCC and cancer-free tissues. NanoString nCounter was used to measure miRNA and mRNA expression and the miRTarBase database was used to validate the predicted miRNA-mRNA interactions.

**Results:**

TargetScan and miRWalk provide a comprehensive overview of potential interactions, whereas miRDB provides functional insights. Our results identified 77 and 154 differentially expressed miRNAs and mRNAs in HNSCC, respectively. miRWalk predicted the highest number of miRNA-mRNA interactions, followed by miRDB and TargetScan. Only 3.3% of interactions were common among the tools. The MiRTarBase analysis confirmed a small subset of the predictions. Biological pathway analysis highlighted the dysregulation of PI3K-Akt and Wnt signaling; miRWalk was the best for elucidating how miRNAs modulate target mRNAs in these key pathways during HNSCC progression.

**Conclusion:**

miRWalk emerged as the most robust tool for predicting miRNA-mRNA interactions. Our findings highlight the importance of integrating bioinformatics predictions with experimental data to better understand the regulatory networks in HNSCC and identify potential biomarkers for diagnosis and therapy.

## In Brief

We evaluated the performance of TargetScan, miRDB, and miRWalk for predicting miRNA-mRNA interactions in HNSCC. Based on clinical tumor and cancer-free tissue data, miRWalk emerged as the most comprehensive tool. Validation using NanoString technology and MiRTarBase confirmed key predictions, highlighting the important roles of the PI3K-Akt and Wnt pathways. This study underscores the importance of integrating bioinformatics and experimental data to better understand HNSCC.

## Highlights

■ miRWalk had the highest predicted interactions and validated miRNA networks in HNSCC.
■ Around 3.3% of interactions overlapped across tools, emphasizing the need for multitool approaches.
■ Dysregulated genes and miRNAs were tied to cancerdriving PI3K-Akt and Wnt pathways.
■ The validated approach highlights the importance of integrating computational and molecular data.

## Graphical abstract

**Figure:**
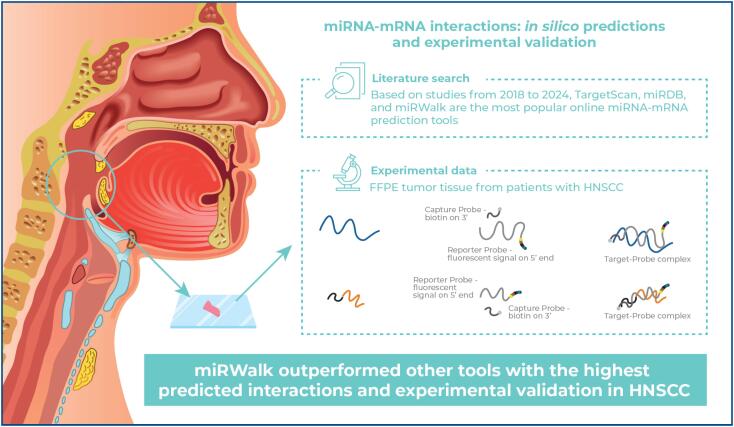

## INTRODUCTION

Head and neck cancer is the seventh most common type of cancer worldwide.^(1)^ In Brazil, >10, 000 deaths were caused by head and neck cancer in 2020, and 22,890 new cases have been predicted for 2023-2025. The disease has a higher prevalence among men, among whom it was anticipated to rank among the top 10 cancers in 2023.^(2)^ Head and neck squamous cell carcinoma (HNSCC) accounts for ~90% of this tumor type.^(3)^ Head and neck squamous cell carcinoma comprises a heterogeneous group of tumors originating in the oral cavity, larynx, nasopharynx, oropharynx, and hypopharynx. It has an estimated 5-year survival rate of 40-50%,^(4)^ primarily due to delays in diagnosis and treatment initiation, which is directly impacted by the lack of specific markers.^(5)^

MicroRNAs (miRNAs) are small non-coding RNA molecules that exert post-transcriptional regulatory functions in conjunction with the RNA-induced silencing complex (RISC). They target the 3’ untranslated region (UTR), 5’ UTR, or promoter region of mRNAs through base pair complementarity.^(6)^ In addition to their biological role, miRNAs are candidate biomarkers owing to their dysregulated expression in tumor tissues; their small size makes them less susceptible to degradation in formalin-fixed paraffin-embedded (FFPE) tissues-the primary sources of clinical specimens.^(7)^ Several studies have suggested that differentially expressed miRNAs act as oncomiRs, tumor suppressors, and prognostic markers in HNSCC.^(8-12)^

Owing to the heterogeneity in HNSCC tumors, there is currently no consensus on effective biomarkers. Further studies are needed to identify diagnostic and prognostic markers and elucidate tumor development, progression, and dynamics.

Identifying the targets of miRNA-mRNA interactions can aid in the discovery of miRNA regulatory networks. In this context, the most effective approach involves computational prediction followed by the validation of miRNA-mRNA interactions. Bioinformatic analyses have shown that a single miRNA can regulate the expression of several genes, and a gene can be controlled by multiple miRNAs. Therefore, the experimental validation of each potential miRNA target is impractical, costly, and time-consuming. Computational approaches for predicting miRNA targets simplify the process by expediting the selection and reducing the number of potential targets for validation.

Several miRNA-mRNA interaction prediction tools are available.^(13)^ As described by Nazarov and colleagues,^(14)^ some use data-driven methods and other target-based methods. Data-driven methods based on similarity use large transcriptomic datasets and advanced methods like correlation analysis and biclustering to accurately identify and prioritize miRNA-mRNA interactions, accounting for complexities and context-specific relationships and improving the accuracy of interaction predictions. Data-driven methods based on matrix factorization integrate mRNA and miRNA expression data by decomposing the expression matrix into lower-rank matrices to capture data variability and identify shared latent variables. Target-based methods can be split into two categories, the first of which is target prediction, such as TargetScan,^(15)^ DIANA-microT-CDS,^(16)^ miRDB,^(17)^ STarMir,^(18)^ miRGator,^(19)^ miRGate,^(20)^ and DeepMirTar.^(21)^ By combining different features and correlations, these tools employ distinct algorithms and methodologies to predict miRNA targets, considering various features such as sequence complementarity, binding site conservation, thermodynamic stability, and experimental validation. Second, various online repositories have been developed for collecting data on experimentally validated interactions between mRNA and miRNAs, such as Diana-Tarbase,^(22)^ miRTarBase,^(23)^ miRecords,^(24)^ and miR2Disease.^(14,25,26)^

In this study, we selected the most commonly used prediction tools to analyze potential miRNA targets in HNSCC. We validated the *in silico* results using molecular barcode hybridization technology (NanoString nCounter) for mRNAs and miRNAs in paired cancer and non-cancer FFPE tissues. This technology is particularly well-suited for situations involving small fragments and a high rate of RNA degradation, such as in FFPE samples. In addition, this platform does not require high computational performance or expertise in data analysis, making it ideal for clinical laboratory applications.

## OBJECTIVE

We aimed to compare the performance of miRNA-mRNA interaction prediction tools, considering both experimental and literature evidence, in accurately representing the characteristics of head and neck squamous cell carcinoma tissues.

## METHODS

### Literature search for miRNA-mRNA interaction prediction tools and comparison using experimental results

We used PubMed Central to search for articles published in English from 2018 to 2024 containing the terms "MicroRNAs," "mRNA-Gene," "Expression Regulation," "carcinoma," "interaction," and "prediction." Tools were chosen based on their online availability and use of an original algorithm or curation method for identifying mRNA-miRNA interactions.

For comparison among tools, we used data on the differentially expressed miRNAs detected between HNSCC and cancer-free tissues, as input. The higher-scoring gene targets for each tool, limited to 10% of the target gene set list (higher scores), were selected and compared with the differentially expressed mRNAs between HNSCC samples and cancer-free samples. Additionally, we also used the MiRTarBase database to search for experimentally validated miRNA-mRNA interactions.

### Study population and sample collection

The cohort comprised 18 patients with a confirmed anatomopathological diagnosis of oropharyngeal squamous cell carcinoma and 9 patients with laryngeal squamous cell carcinoma (Table 1). The average patient age was 58 years (range, 39-85 years) and the male/female ratio was approximately 4:1. Most of the patients were smokers or former smokers (Table 1). All samples were obtained from patients treated at the *Instituto de Cancer, Dr. Arnaldo Vieira de Carvalho* (ICAVC, São Paulo, Brazil). Clinical TNM classification was performed according to the Union for International Cancer Control staging classification system for HNSCC.^(27)^ This study was approved by the Institutional Review Board of *Hospital Israelita Albert Einstein* (CAAE: 94534318.3.0000.0071; # 3.780.449), and written informed consent was obtained from all participants. Analysis of hematoxylin and eosin (H&E)-stained sections was performed by expert pathologists, who confirmed the presence of ≥50% tumor cells in all HNSCC samples.

**Table 1:** Clinical and pathological characteristics of the study group

Patient ID	Age	Sex	TNM clinical classification	Site	Smoking status	Alcohol consumption	Assay
A1	50	Female	T4N1M0	Larynx	No	No	miRNA
A2	48	Male	T1N0M0	Oropharynx	Yes	Former	PanCancer and miRNA
A3	54	Male	T4N0M0	Larynx	Yes	Yes	miRNA
A4	69	Male	T2N2aM0	Larynx	Former	Former	miRNA
A5	43	Male	T2N3bM0	Oropharynx	Yes	Former	PanCancer and miRNA
A6	45	Male	T1N2M0	Oropharynx	No	No	PanCancer and miRNA
A7	46	Female	T1N2M0	Oropharynx	No	No	PanCancer and miRNA
A8	56	Female	T2N0M0	Oropharynx	Yes	No	PanCancer and miRNA
A9	67	Male	T2N0M0	Oropharynx	Former	Former	PanCancer and miRNA
A10	68	Male	T3N0M0	Larynx	Yes	Yes	miRNA
A11	39	Male	T2N2aM0	Larynx	Former	Yes	miRNA
A12	41	Male	T3N1M0	Oropharynx	Former	Former	miRNA
B1	67	Male	T4N2cM0	Larynx	Former	Former	miRNA
B2	69	Male	T4N0M0	Oropharynx	Yes	Yes	miRNA
B3	56	Male	T4N0M0	Larynx	Yes	Yes	miRNA
B4	60	Male	T3N1M0	Oropharynx	Former	Former	PanCancer
B5	48	Male	T2N1M0	Oropharynx	Yes	Former	miRNA
B6	54	Male	T4aN0M0	Oropharynx	Yes	Former	PanCancer
B7	69	Male	T2N2bM0	Oropharynx	Former	Former	PanCancer
B8	77	Female	T4N2bM0	Larynx	-	-	miRNA
B9	45	Male	T4aN2bM0	Oropharynx	Yes	Yes	PanCancer
B10	57	Male	T3N2aM0	Oropharynx	Yes	Former	PanCancer
B11	63	Male	T4N2bM0	Oropharynx	Former	Former	miRNA
B12	67	Male	T4N2cM0	Oropharynx	Yes	Former	miRNA
B13	85	Male	T4aN0M0	Larynx	Former	Former	miRNA
B14	70	Female	T4N2aM0	Oropharynx	Former	Former	miRNA
B15	59	Male	T4aN3M0	Oropharynx	Yes	Former	PanCancer

### RNA isolation

We used 21 tissue samples from patients with HNSCC (12 oropharynx and 9 larynx) for miRNA profiling, and 12 HNSCC samples were used for mRNA expression analysis. Formalin-fixed paraffin-embedded tissue blocks were cut into 10μm-thick sections using a microtome and adhered to glass slides. Based on the H&E-stained slides, tumor and non-tumor areas were separately scraped using a scalpel and loaded into a 1.5mL tube. Total RNA, including miRNA, was isolated from FFPE tissues using an AllPrep DNA/RNA FFPE kit (Qiagen, Hilden, Germany) according to the manufacturer's protocol.

### miRNA expression

An nCounter Human v3 miRNA Expression Assay (NanoString Technologies, Seattle, WA, USA) was used for the miRNA profiling of 21 tissue samples from patients with HNSCC (n=12 oropharynx and 9 larynx) using the total RNA isolated from HNSCC and cancer-free FFPE tissues (60-100ng), according to the manufacturer's recommendations. Each cartridge was scanned with a 555 FOV using an nCounter Digital Analyzer. Data processing was performed using nSolver Analysis Software 4.0 (NanoString Technologies). Background subtraction was performed using the geometric mean of the negative controls plus one standard deviation, normalization to the geometric mean of the positive controls, and normalization to the geometric mean of the top 100 most highly expressed miRNAs. After normalization, we filtered miRNAs with fewer than three counts from >30% of the samples.

### mRNA expression

For mRNA, tumor and non-tumor FFPE tissue RNA from 12 patients with oropharyngeal squamous cell carcinoma were analyzed using the nCounter PanCancer Pathways Panel (NanoString Technologies). This tool offers fast and high-throughput multiplex capabilities for analyzing human gene expression. Each panel comes with 770 genes from 13 cancer-associated canonical pathways representing the basic cancer biology [Notch, MAPK, STAT, PI3K, RAS, cell cycle, apoptosis, Hedgehog, APC (Wnt), DNA damage and repair control, transcriptional misregulation, chromatin modification, and TGF-β]. We used ~300ng RNA in the assay, according to the manufacturer's recommendations. Samples with <100 counts for 100% of the target mRNAs were excluded from further analysis. Data were normalized using the geNorm algorithm, which selects the most stable housekeeping genes that exhibit the least variation among the samples.

### Statistical analysis

A two-tailed *t*-test was used to assess the miRNA counts obtained using the nCounter Human v3 assay and mRNA counts derived using the nCounter PanCancer Pathways Panel. nSolver Analysis Software 4.0 was used for statistical analysis, with significance set at p≤0.05 for miRNAs and p<0.05 for mRNAs. Adjusted p-values corrected for multiple testing using the Benjamini-Yekutieli method (assigned as FDR) were estimated for genes involved in key biological processes.

## RESULTS

### Most commonly used miRNA-mRNA interaction tools

**Figure 1:**
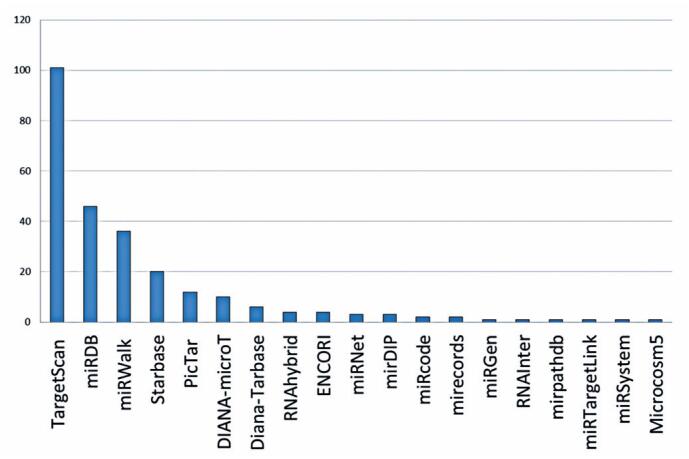
Most used miRNA-mRNA interaction tools from PubMed search for 2018-2024. The x-axis lists the tools that met the study inclusion criteria and y-axis shows the number of publications utilizing each tool

Based on the literature search and accounting for the limitations of the keywords, a total of 247 articles were identified, of which 150 matched our inclusion criteria (Figure 1).

Of the 150 studies, 67% used TargetScan 8.0, 46% miRDB, and 36% miRWalk 2.0. Thus, we selected these tools for our prediction analysis and used MiRTarBase to validate the predicted interactions.

TargetScan 8.0^(17)^ is the most widely used miRNA-mRNA interaction prediction tool, with the current version available since 2021. This tool predicts miRNA targets based on seed region complementarity and evolutionary conservation by applying a context score that reflects the potential of a miRNA-binding site for creating an effective miRNA-mRNA interaction, considering 14 parameters accounting for interactions in canonical and non-canonical sites.

The miRWalk 2.0 database^(28)^ contains results from the TarPmiR algorithm,^(29)^ a random forest-based approach that predicts miRNA-mRNA interactions through the use of "decision trees" with set rules for decision making. It is similar to a fluxogram with "knots" where, if the condition is verified, flow is continued in the same direction, otherwise a different direction is taken towards the next knot. This approach integrates key features such as accessibility, seed match, flanking conservation, folding energy, the length of the largest consecutive pairings, the length of the target site, AU content, stem conservation, difference between stem and flanking conservation, m/e motif, and difference between the numbers of paired positions in the seed region and miRNA 3’ end to predict miRNA target sites.^(29)^

miRDB^(17)^ is another online database that uses the machine learning algorithm MirTarget^(30)^ for target prediction. Together with the more commonly used miRNA-targeting features used for predicting interactions, this tool combines data from miRNA overexpression and crosslinking and immunoprecipitation binding experiments data to identify transcript targets associated with functional RISC complexes, providing a functional weight for the predictions.

The miRTarBase database^(22)^ relies on experimentally validated interactions, curated data from the literature, and high-throughput experiments. miRTarBase includes interactions confirmed through methods such as reporter assays (western blotting, microarray experiments, and high-throughput sequencing). The database is compiled of data from manually reviewed scientific articles reporting validated miRNA-target interactions.

### miRNAs and mRNAs expressed in HNSCC

**Figure 2:**
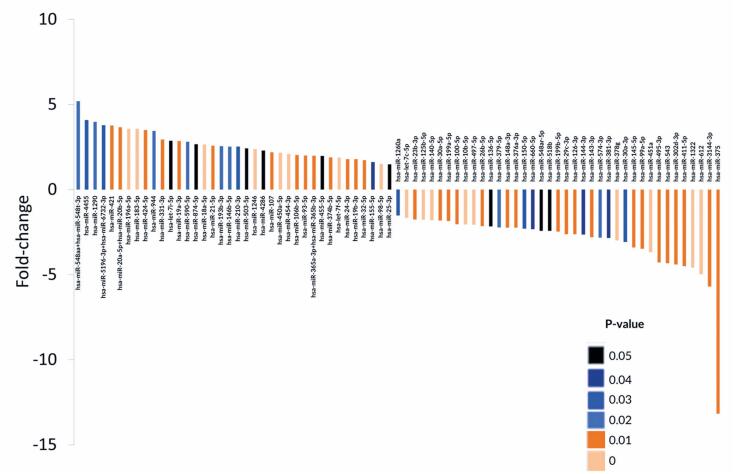
MicroRNAs differentially expressed between tumor and non-tumor tissues (p≤0.05). Positive and negative fold-change describe upregulated and downregulated miRNAs in tumor tissue, respectively

We identified 77 miRNAs that were differentially expressed between cancer and cancer-free areas; 38 and miRNAs were upregulated and downregulated in tumor tissues, respectively (Figure 2). For mRNAs, 154 were differentially expressed between cancer and cancer-free tissues; 87 were upregulated and 67 were downregulated in tumor tissues. Among the differentially expressed genes, 15 were previously reported to be dysregulated in HNSCC (Table 2). These genes participate in the cell cycle, apoptosis, and PI3K, Wnt, and Hedgehog pathways (Table 2).

**Table 2:** Genes differentially expressed (p<0.05) in tumor tissue and previously described for HNSCC

Gene	Log2 fold-change tumor *versus* non-tumor	Pathway	Gene	Log2 fold-change tumor *versus* non-tumor	Pathway
LAMC2	3.47	PI3K	WNT4	1.44	Hedgehog, Wnt
E2F1	3.05	Cell Cycle - Apoptosis	SKP2	1.42	Cell Cycle - Apoptosis
PKMYT1	2.92	Cell Cycle - Apoptosis	CDK6	1.29	Cell Cycle - Apoptosis, PI3K
WNT7B	2.52	Hedgehog, Wnt	ITGB4	1.22	PI3K
MCM2	2.32	Cell Cycle - Apoptosis	ITGA6	1.15	PI3K
CDC6	2.25	Cell Cycle - Apoptosis	ITGA3	1.12	PI3K
LAMB3	1.95	PI3K	CDKN1C	-1.07	Cell Cycle - Apoptosis
CCNA2	1.68	Cell Cycle - Apoptosis			

### miRNA-mRNA interaction predictions and biological implications

**Figure 3:**
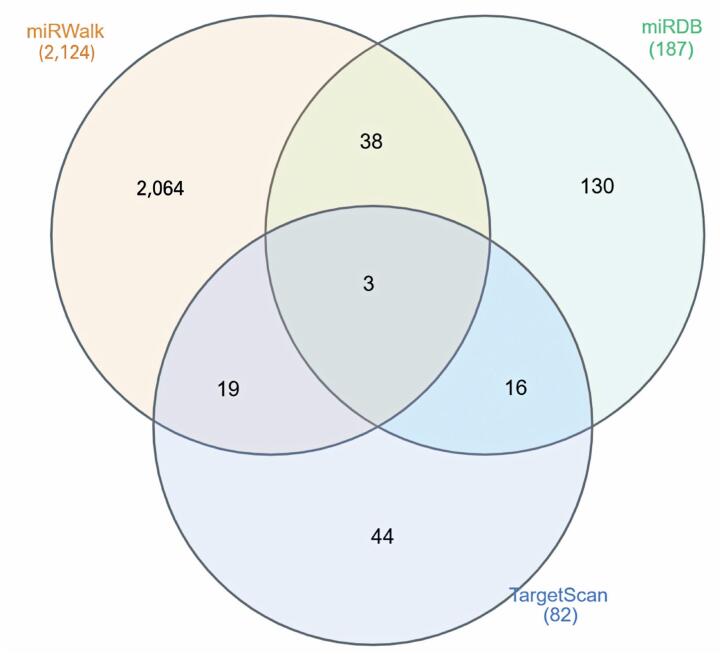
Differentially expressed genes targeted by differentially expressed miRNAs between tumor and tumor-free tissues. MiRWalk, miRDB, and TargetScan databases were used to predict miRNA targets. The numbers of miRNA-mRNA interactions are presented in the Venn diagram

We evaluated the potential mRNA targets of the differentially expressed miRNAs using the different prediction tools. Using the 77 differentially expressed miRNAs as input, we identified interactions with 154 differentially expressed mRNAs in tumor tissues: 2,124 interactions using miRWalk, 187 using miRDB, and 82 using TargetScan (Figure 3), of which 42, 9, and 17 were validated using MiRTarBase; however, none were previously associated with HNSCC. Notably, one miRNA can regulate multiple target genes, whereas a single gene can be regulated by several miRNAs.^(31)^

Only 3.3% (76/2,314) of the interactions were shared among at least two of the prediction tools. Only 2.4% (59/2,463) of the predicted interactions were validated using MiRTarBase. Although the three tools predicted three common interactions (Figure 3), these were not consistent with the experimentally validated database.

**Figure 4:**
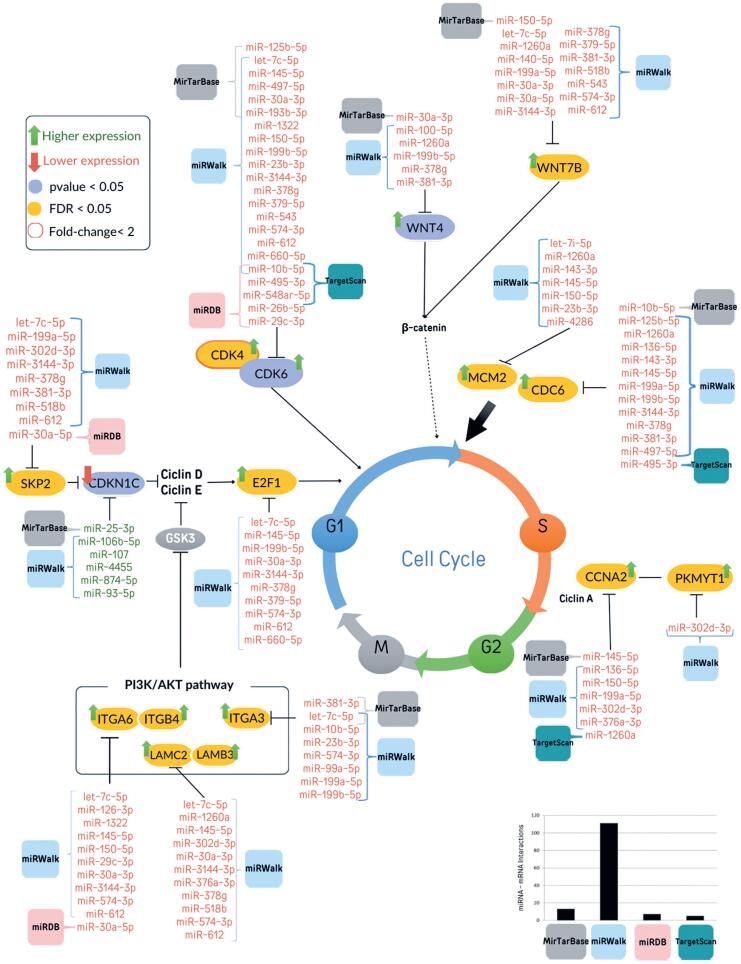
Proposed model of how differently expressed mRNA and miRNA may contribute to tumor progression. The histogram represents the number of interactions predicted by each of the tools for differentially expressed mRNA and miRNA. miRNAs in red and green were downregulated and upregulated in tumor tissue, respectively. Green and red arrows represent upregulated and downregulated genes in tumor tissue, respectively. Genes in blue were differentially expressed (p<0.05). Genes in yellow had an adjusted p<0.05 (FDR)

Based on our data and the literature, the altered expression of mRNAs in this cohort promotes tumor progression by enhancing proliferative signals and PI3K-Akt and WNT pathway activities. Figure 4 illustrates these alterations based on differentially expressed miRNAs and target genes predicted using at least one of the four tools. In tumor tissues, we identified the upregulation of genes involved in activating the cell cycle and DNA replication, and downregulation of miRNAs predicted to target these genes. miRWalk predicted the most interactions between miRNAs and mRNAs, followed by miRDB and TargetScan, with 111, seven, and five interactions, respectively (Figure 4).

## DISCUSSION

Several miRNA-mRNA interaction prediction tools have been developed, each applying distinct algorithms and methodologies to predict miRNA targets. These algorithms consider various features, such as sequence complementarity, binding site conservation, thermodynamic stability, and experimental validation, to promote the accuracy of these tools.

Although TargetScan, miRDB, and miRWalk share similar features^(32)^ (such as sequence complementarity, total number of paired positions, target site accessibility, and conservation), only ~3% of the miRNA-mRNA interactions was predicted by more than one of them. This is consistent with the 13% similarity previously reported between miRDB and TargetScan.^(14)^ In another study, in which 10 of the top miRNAs with MiRTarBase-validated targets were analyzed using TargetScan, miRanda-mirSVR, Pita, and RNA22, 18% of the validated interactions were not predicted by any tool.^(33)^ This suggests that biological processes may not be fully captured by prediction tools.

The best prediction tool in our study was miRWalk, since it had the highest numbers of predicted and validated interactions. Similarly, a previous study reported better performance for miRWalk among several prediction tools.^(29)^ miRWalk also shared the most interactions with the other tools.

Despite the low similarity between the different tools, they are the most commonly used among researchers in this field. This could be because of their continued updates and community involvement, whereas many other tools have been discontinued.^(34)^ In terms of publication count, TargetScan was the most common, possibly because of the amount of information it provides and its pioneering role in predicting miRNA targets in vertebrate research.^(35)^ In our study, TargetScan identified the fewest validated interactions, likely because of its high false negative rate and stringent criteria for interaction sites.^(36)^ These criteria require high confidence, canonical interactions primarily within the 3’-UTR, and evolutionary conservation. In contrast, miRWalk is more flexible and inclusive, broadening the range of potential interactions to those with mismatches or noncanonical binding patterns across the entire mRNA. Notably, our analysis was limited to the top-scoring interactions from all four tools, which may have contributed to the lower perceived sensitivity of TargetScan.

The nCounter PanCancer Pathways Panel allowed us to identify differences in the expression of genes related to commonly dysregulated pathways in cancer. Genes involved in the PI3K pathway, which are crucial for cellular functions such as transcription, translation, proliferation, and survival, are often overexpressed in HNSCC.^(37)^ We identified several candidate miRNA-mRNA interactions that should be investigated as targets in HNSCC. Notably, integrin subunit alpha 3 (ITGA3) and ITGA6 showed significantly increased expression in patients with HNSCC, with higher levels of ITGA3 linked to poorer overall and relapse-free survival rates.^(38)^ We observed the downregulation of miR-150-5p and upregulation of ITGA6 in tumor tissues, consistent with the findings of Koshizuka et al.^(38)^ This interaction was predicted only by miRWalk. Although it uses a combination of manual curation and machine learning methods to validate interactions with high accuracy (>82%), MiRTarBase did not capture this interaction, indicating possible limitations in curation.

ITGB4 is upregulated in primary and metastatic HNSCC, associated with poor prognosis, and its knockdown reduces EGFR-mediated migration and invasion.^(39,40)^ Overexpression of ITGA6 and laminin subunit gamma 2 (LAMC2) was previously reported in HNSCC; LAMC2 has been associated with poor prognosis and identified as a potential biomarker for cancer invasion.^(41-44)^ Laminin subunit beta 3 (LAMB3) enhances cisplatin sensitivity and reduces cell migration and invasion.^(45,46)^ In our study, these genes were upregulated in tumor tissues and miRNAs predicted to interact with these genes were identified as less expressed in the tumor tissue. Collectively, these findings support the role of these genes in HNSCC tumorigenesis and progression.

Overactivation of WNT-β-catenin signaling promotes cancer cell proliferation and survival.^(47)^ Consistent with our findings, Wnt family member 7 B (WNT7B) is upregulated in oral squamous cell carcinoma (OSCC), and *in vitro* knockdown of WNT7B in OSCC cell lines inhibits cell invasion.^(48)^ Overexpression of WNT4 in laryngeal cancer cell lines promoted the nuclear accumulation of β-catenin while knockdown of WNT4 had the opposite results.^(49)^ In our study, these genes were upregulated in tumor tissues corroborating the literature, while miRNAs predicted to have these genes as targets were found with less expression in tumor tissue, such as miR-30a and miR-150.

Genes that regulate the cell cycle and apoptosis are crucial for tumor progression and cell proliferation. In our tumor samples, we observed the overexpression of S-phase kinase-associated protein 2 (SKP2) and downregulation of cyclin-dependent kinase inhibitor 1C (CDKN1C). Notably, SKP2 expression has been correlated with shorter overall survival in patients with HNSCC^(50)^ and is often inversely associated with CDKN1C expression in various cancers.^(51,52)^ In OSCC, positive CDKN1C staining was correlated with higher 5-year survival rates. Additionally, CDKN1C mRNA expression decreased from leukoplakia with moderate or severe dysplasia to OSCC.^(53)^ Consistent with other studies, we observed the upregulation of protein kinase membrane-associated tyrosine/threonine 1 (PKMYT1), cyclin A2 (CCNA2), minichromosome maintenance complex component 7 (MCM7), cell division cycle 6 (CDC6),^(54-56)^ cyclin-dependent kinase 4 (CDK4), and CDK6, which are potential immunotherapy targets in HNSCC.^(57,58)^ Additionally, we observed reduced expression of miRNAs (such as miR-145, miR-30a-3p, and let-7c-5p) predicted by miRWalk to target CDK6 and we found these interactions validated in miRTarBase database.^(59-61)^ Further research into these regulatory networks may unveil new therapeutic strategies and biomarkers for the better management and prognosis of this challenging disease.

Changes in the cellular phenotype of tumor cells reflect changes in the expression of several genes caused by different carcinogenic and/or biological processes. Here, we hypothesized that differences in mRNA expression between tumor and non-tumor tissues may be partially caused by differences in miRNA expression, as supported by the miRNA-mRNA interaction tools. Our study provides useful information for elucidating the mechanisms of tumor progression in HNSCC, especially given the current lack of biomarkers.

In summary, depending on the research aim, our findings demonstrated the effective use of TargetScan and miRWalk for gaining a broad overview of potential interactions, miRDB for gaining functional insights, and miRTarBase for validation. Although the focus of miRTarBase on experimentally validated interactions may enhance reliability, it may limit the scope of the results. Combining these four tools may address some of their separate limitations while complementing their strengths. Although these methods are suitable for preliminary investigations, the predictions should be supplemented with experimentally validated data to ensure biological relevance. Through experimental validation, we identified miRWalk as the most appropriate tool for assessing how miRNAs modulate differentially expressed target mRNAs in HNSCC, followed by miRDB and TargetScan. Considering the limitations of each database, miRWalk enables comprehensive exploration by integrating data from the other three databases, offering a well-rounded approach to studying miRNA interactions.

## CONCLUSION

Databases and online tools provide excellent resources for the initial review and identification of miRNA and mRNA interactions. However, their output must be interpreted cautiously, considering the limitations of each method.
